# Inference of Genetic Networks From Time-Series and Static Gene Expression Data: Combining a Random-Forest-Based Inference Method With Feature Selection Methods

**DOI:** 10.3389/fgene.2020.595912

**Published:** 2020-12-15

**Authors:** Shuhei Kimura, Ryo Fukutomi, Masato Tokuhisa, Mariko Okada

**Affiliations:** ^1^Faculty of Engineering, Tottori University, Tottori, Japan; ^2^Graduate School of Sustainability Science, Tottori University, Tottori, Japan; ^3^Laboratory of Cell Systems, Institute of Protein Research, Osaka University, Osaka, Japan

**Keywords:** FANTOM5, gene expression, feature selection, random forest, genetic network inference

## Abstract

Several researchers have focused on random-forest-based inference methods because of their excellent performance. Some of these inference methods also have a useful ability to analyze both time-series and static gene expression data. However, they are only of use in ranking all of the candidate regulations by assigning them confidence values. None have been capable of detecting the regulations that actually affect a gene of interest. In this study, we propose a method to remove unpromising candidate regulations by combining the random-forest-based inference method with a series of feature selection methods. In addition to detecting unpromising regulations, our proposed method uses outputs from the feature selection methods to adjust the confidence values of all of the candidate regulations that have been computed by the random-forest-based inference method. Numerical experiments showed that the combined application with the feature selection methods improved the performance of the random-forest-based inference method on 99 of the 100 trials performed on the artificial problems. However, the improvement tends to be small, since our combined method succeeded in removing only 19% of the candidate regulations at most. The combined application with the feature selection methods moreover makes the computational cost higher. While a bigger improvement at a lower computational cost would be ideal, we see no impediments to our investigation, given that our aim is to extract as much useful information as possible from a limited amount of gene expression data.

## 1. Introduction

The dynamic behavior of gene expression determines a variety of cell functions. Our understanding of biological systems requires the study of complex patterns of gene regulation, as the regulation among genes determines how genes are expressed. One promising approach developed for the analysis of gene regulation is the inference of genetic networks. In a genetic network inference problem, mutual regulations among genes are inferred from gene expression data measured by biological technologies, such as DNA microarrays, RNA-seq using next generation sequencers, and so on. The inferred network models can serve as ideal tools to help biologists generate hypotheses and facilitate the design of their experiments. Many researchers have thus taken an interest in the inference of genetic networks.

A number of genetic network inference methods have been proposed (Larrañaga et al., 2006; Meyer et al., 2008; Chou and Voit, 2009; Hecker et al., 2009; de Matos Simoes and Emmert-Streib, 2012; Emmert-Streib et al., 2012; Glass et al., 2013). Among them, random-forest-based methods show promise for their excellent performance (Huynh-Thu et al., 2010; Maduranga et al., 2013; Petralia et al., 2015; Huynh-Thu and Geurts, 2018; Kimura et al., 2019). Some of these inference methods also have a useful ability to analyze both time-series and static gene expression data (Petralia et al., 2015; Huynh-Thu and Geurts, 2018; Kimura et al., 2019). The time-series data are a series of sets of gene expression levels measured at successive time points after a stimulation. The static data are sets of gene expression levels measured under steady-state conditions. The random-forest-based inference methods analyze gene expression data by assigning confidence values to all of the candidate regulations. While many genetic network inference methods try to find regulations that are actually contained in the target network, the random-forest-based methods only rank the candidates by assigning every candidate a confidence value. When biologists try to perform experiments for confirming the inferred regulations of genes, the confidence values computed by the random-forest-based methods could be used to determine the order of the experiments. The random-forest-based inference methods would become much more useful, however, if they had the ability to detect genes that actually regulated a gene of interest.

By combining the random-forest-based inference method with some feature selection method, we have been able to detect regulations that are actually contained in the target genetic network. Feature selection, a procedure studied in the computational intelligence field, removes input variables irrelevant to the output in an approximation task or a classification task (Guyon and Elisseeff, 2003; Cai et al., 2018). We found, however, in preliminary experiments, that a combined method integrating the random-forest-based method with one of the existing feature selection methods often fails to detect genes that weakly affect a gene of interest. The main purpose of the existing feature selection methods might explain this failure, as the methods were developed not to detect all of the input variables that actually affect the output, but to find input variables that maximize the predicting performance of the obtained model. More recently, our group developed a new feature selection method whose purpose is to find all of the input variables that actually affect the output and to remove as many of the irrelevant input variables as possible (Kimura and Tokuhisa, 2020).

In this manuscript, we propose a method to remove unpromising candidate regulations by combining the random-forest-based inference method with the new feature selection method we developed in Kimura and Tokuhisa (2020), along with two modified versions of the same. The feature selection methods used in this study are effective in not only removing several irrelevant input variables, but also in assigning confidence values to the input variables to show the likelihood that they actually affects the output. In our combined method, we can therefore use the confidence values computed by the feature selection methods to adjust the confidence values assigned to all of the candidate regulations by the random-forest-based method.

The remainder of this manuscript is organized as follows. In the section 2, we introduce the random-forest-based inference method used in this study. In the section 3, we describe the feature selection methods, and then explain a way to combine them with the inference method. We confirm the effectiveness of the proposed combined method through numerical experiments using artificial and biological gene expression data in the sections 4 and 5, respectively. Finally, in the section 6, we conclude with our future work.

## 2. Random-Forest-Based Inference Method

As mentioned previously, this study combines the random-forest-based inference method with a series of feature selection methods. While any random-forest-based method can serve this purpose, in this study we apply an inference method (Kimura et al., 2019) that is capable of analyzing both time-series and static gene expression data. This section briefly describes the inference method.

### 2.1. Model for Describing Genetic Networks

The inference method applied in this study describes a genetic network using a set of differential equations of the form

(1) $$\begin{array}{l} \frac{dX_{n}}{dt}=F_{n}\left({\text{X}}_{-n}\right)-\beta_{n}X_{n},\;(n=1,2,\cdots \;,N), \end{array}$$

where ***X***_−*n*_ = (*X*_1_, ⋯ , *X*_*n*−1_, *X*_*n*+1_, ⋯ , *X*_*N*_), *X*_*m*_ (*m* = 1, 2, ⋯ , *N*) is the expression level of the *m*-th gene, *N* is the number of genes contained in the target network, β_*n*_ (> 0) is a constant parameter, and *F*_*n*_ is a function of arbitrary form.

When using this model, we infer a genetic network by obtaining a function *F*_*n*_ and a parameter β_*n*_ (*n* = 1, 2, ⋯ , *N*) that produce time-courses consistent with the observed gene expression levels. The following section presents a way to obtain them.

### 2.2. Obtaining *F*_*n*_ and β_*n*_

The inference method (Kimura et al., 2019) divides an inference problem of a genetic network consisting of *N* genes into *N* subproblems, each of which corresponds to each gene. By solving the *n*-th subproblem, the method obtains a reasonable approximation of the function *F*_*n*_ and a reasonable value for the parameter β_*n*_. The remainder of this section will describe the *n*-th subproblem.

#### 2.2.1. Problem Definition

The inference method used in this study obtains an approximation of the function *F*_*n*_ and a value for the parameter β_*n*_ through the optimization of the following one-dimensional function.

(2) $$\begin{array}{l} S_{n}\left(\beta_{n}\right)=\overset{K_{T}}{\underset{k=1}{\sum }}\frac{w_{k}^{T}}{\beta_{n}}{\left[\frac{dX_{n}}{dt}{|}_{t_{k}}-{\hat{F}}_{n}\left({\text{X}}_{-n}{|}_{t_{k}};\beta_{n}\right)+\beta_{n}X_{n}{|}_{t_{k}}\right]}^{2}\\ \;+\overset{K_{S}}{\underset{k=1}{\sum }}\frac{w_{k}^{S}}{\beta_{n}}{\left[\frac{dX_{n}}{dt}{|}_{s_{k}}-{\hat{F}}_{n}\left({\text{X}}_{-n}{|}_{s_{k}};\beta_{n}\right)+\beta_{n}X_{n}{|}_{s_{k}}\right]}^{2}, \end{array}$$

where ***X***_−*n*_|_*t*_*k*__ = (*X*_1_|_*t*_*k*__, ⋯ , *X*_*n*−1_|_*t*_*k*__, *X*_*n*+1_|_*t*_*k*__, ⋯ , *X*_*N*_|_*t*_*k*__), ***X***_−*n*_|_*s*_*k*__ = (*X*_1_|_*s*_*k*__, ⋯ , *X*_*n*−1_|_*s*_*k*__, *X*_*n*+1_|_*s*_*k*__, ⋯ , *X*_*N*_|_*s*_*k*__), and *X*_*m*_|_*t*_*k*__ and *X*_*m*_|_*s*_*k*__ (*m* = 1, 2, ⋯ , *N*) are the expression levels of the *m*-th gene at the *k*-th measurement in time-series and steady-state experiments, respectively. *K*_*T*_ (≥ 2) and *K*_*S*_ (≥ 0) are the numbers of measurements performed in the time-series and steady-state experiments, respectively. Note that the expression levels *X*_*m*_|_*t*_*k*__ and *X*_*m*_|_*s*_*k*__ are measured using biochemical techniques in the genetic network inference. $\frac{dX_{n}}{dt}{|}_{t_{k}}$ and $\frac{dX_{n}}{dt}{|}_{s_{k}}$ are the time derivatives of the expression level of the *n*-th gene at the *k*-th measurement in the time-series and steady-state experiments, respectively. The time derivatives of the expression level of the *n*-th gene in the time-series experiments, i.e., $\frac{dX_{n}}{dt}{|}_{t_{k}}$'s, are directly estimated from the measured time-series of the gene expression levels using a smoothing technique, such as a spline interpolation (Press et al., 1995), a local linear regression (Cleveland, 1979), a modified Whittaker's smoother (Vilela et al., 2007), or the like. On the other hand, the time derivatives of the expression level of the *n*-th gene in the steady-state experiments, i.e., $\frac{dX_{n}}{dt}{|}_{s_{k}}$'s, are all set to zero. $w_{k}^{T}$ and $w_{k}^{S}$ are weight parameters for the *k*-th measurements in the time-series and steady-state experiments, respectively. Kimura et al. (2019) showed that the performance of the random-forest-based inference method improves by discounting the weight values of the measurements that were obtained under states similar to each other. ${\hat{F}}_{n}\left(\cdot ;\beta_{n}\right)$ is an approximation of the function *F*_*n*_ trained under the given β_*n*_. The inference method (Kimura et al., 2019) obtains an approximation of the function *F*_*n*_ using a random forest (Breiman, 2001). The section 2.2.2 below will describe a way to obtain ${\hat{F}}_{n}$ using a random forest. The inference method described here uses the golden section search (Press et al., 1995) to minimize the objective function (2).

#### 2.2.2. Approximation of *F*_*n*_

The computation of the objective function (2) requires an approximation of the function *F*_*n*_, i.e., ${\hat{F}}_{n}$. As described previously, an approximation of the function *F*_*n*_ is obtained using a random forest. In the inference method (Kimura et al., 2019), the random forest that approximates the function *F*_*n*_ is trained based on training data consisting of the following set of input-output pairs,

$$\begin{array}{l} \left\{\left({\text{X}}_{-n}{|}_{t_{k}},\frac{dX_{n}}{dt}{|}_{t_{k}}+\beta_{n}X_{n}{|}_{t_{k}}\right)|k=1,2,\cdots \;,K_{T}\right\}\\ \;\cup \left\{\left({\text{X}}_{-n}{|}_{s_{k}},\frac{dX_{n}}{dt}{|}_{s_{k}}+\beta_{n}X_{n}{|}_{s_{k}}\right)|k=1,2,\cdots \;,K_{S}\right\}. \end{array}$$

Note that a value for the parameter β_*n*_ is always given when computing a value for the objective function (2). Therefore, we can train the random forest using the training data described above. Note also that, in order to keep consistency with the objective function (2), the random forest used in the method (Kimura et al., 2019) tries to obtain an approximation of the function *F*_*n*_ that minimizes a weighted sum of the squared errors between the given output values and the values computed from the model.

### 2.3. Assigning Confidence Values to the Regulations

As is done in other random-forest-based inference methods, the inference method described in this section uses a variable importance measure defined in tree-based machine learning techniques, such as a random forest, to evaluate the confidence values of all of the candidate regulations. Note again, however, that the random forest used in the inference method tries to minimize the weighted sum of the squared errors. The confidence value of the regulation of the *n*-th gene from the *m*-th gene, *C*_*n,m*_, is thus computed by

(3) $$\begin{array}{l} C_{n,m}=\frac{1}{Sq_{w0}}\frac{1}{N_{tree}}\overset{N_{tree}}{\underset{i=1}{\sum }}\underset{\nu \in V_{i}\left(m\right)}{\sum }I\left(\nu \right), \end{array}$$

where

(4) $$\begin{array}{l} Sq_{w0}=\overset{K_{T}}{\underset{k=1}{\sum }}w_{k}^{T}{\left(y_{t_{k}}-{\bar{y}}_{w0}\right)}^{2}+\overset{K_{S}}{\underset{k=1}{\sum }}w_{k}^{S}{\left(y_{s_{k}}-{\bar{y}}_{w0}\right)}^{2}, \end{array}$$

(5) $$\begin{array}{l} {\bar{y}}_{w0}=\frac{1}{N_{w0}}\left[\overset{K_{T}}{\underset{k=1}{\sum }}w_{k}^{T}y_{t_{k}}+\overset{K_{S}}{\underset{k=1}{\sum }}w_{k}^{S}y_{s_{k}}\right], \end{array}$$

(6) $$\begin{array}{l} N_{w0}=\overset{K_{T}}{\underset{k=1}{\sum }}w_{k}^{T}+\overset{K_{S}}{\underset{k=1}{\sum }}w_{k}^{S}, \end{array}$$

(7) $$\begin{array}{l} y_{t_{k}}=\frac{dX_{n}}{dt}{|}_{t_{k}}+\beta_{n}^{*}X_{n}{|}_{t_{k}}, \end{array}$$

(8) $$\begin{array}{l} y_{s_{k}}=\frac{dX_{n}}{dt}{|}_{s_{k}}+\beta_{n}^{*}X_{n}{|}_{s_{k}}, \end{array}$$

(9) $$\begin{array}{l} I\left(\nu \right)=N_{w}\left(\nu \right)Sq_{w}\left(\nu \right)-N_{w}\left(\nu_{L}\right)Sq_{w}\left(\nu_{L}\right)\\ \;-N_{w}\left(\nu_{R}\right)Sq_{w}\left(\nu_{R}\right), \end{array}$$

(10) $$\begin{array}{l} Sq_{w}\left(\nu \right)=\underset{k\in T\left(\nu \right)}{\sum }w_{k}^{T}{\left[y_{t_{k}}-{\bar{y}}_{w}\left(\nu \right)\right]}^{2}\\ \;+\underset{k\in S\left(\nu \right)}{\sum }w_{k}^{S}{\left[y_{s_{k}}-{\bar{y}}_{w}\left(\nu \right)\right]}^{2}, \end{array}$$

(11) $$\begin{array}{l} {\bar{y}}_{w}\left(\nu \right)=\frac{1}{N_{w}\left(\nu \right)}\left[\underset{k\in T\left(\nu \right)}{\sum }w_{k}^{T}y_{t_{k}}+\underset{k\in S\left(\nu \right)}{\sum }w_{k}^{S}y_{s_{k}}\right], \end{array}$$

(12) $$\begin{array}{l} N_{w}\left(\nu \right)=\underset{k\in T\left(\nu \right)}{\sum }w_{k}^{T}+\underset{k\in S\left(\nu \right)}{\sum }w_{k}^{S}, \end{array}$$

*N*_*tree*_ is the number of trees in the random forest ${\hat{F}}_{n}^{*}$, and *V*_*i*_(*m*) is a set of nodes that use the expression levels of the *m*-th gene to split the training examples in the *i*-th decision tree of ${\hat{F}}_{n}^{*}$. ν_*L*_ and ν_*R*_ are the left and right children nodes of the node ν, respectively, and *T*(ν) and *S*(ν) are sets of indices of the training examples generated from time-series and static gene expression data, respectively, and allocated to the node ν. ${\hat{F}}_{n}^{*}$ and $\beta_{n}^{*}$ are the approximation of the function *F*_*n*_ and the value for the parameter β_*n*_, respectively, obtained through the optimization of the function (2).

## 3. Combining a Random-Forest-Based Inference Method With Feature Selection Methods

As mentioned previously, any existing feature selection method can be combined with a random-forest-based inference method. We found however that the combination of the random-forest-based method and an existing feature selection method often degrades the quality of the inferred genetic network. This degradation might be explained by the purpose for which the existing feature selection methods were designed, namely, to select input variables that maximize the predicting performance of the approximated function. More recently, however, Kimura and Tokuhisa (2020) proposed a new feature selection method that seeks to find all of the input variables that actually affect the output. In this study, we combine the random-forest-based inference method described in the previous section with this new feature selection method (Kimura and Tokuhisa, 2020), along with two modified versions of the same.

In this section, we first describe the new feature selection method (Kimura and Tokuhisa, 2020) as originally proposed and several modified forms, and then propose a way to combine them with the random-forest-based inference method.

### 3.1. Feature Selection Methods Based on Variable Importance Measure

The feature selection method (Kimura and Tokuhisa, 2020), we apply uses a variable importance measure to check whether or not each input variable actually affects the output. If a certain input variable is relevant to the output, its importance score is likely to be larger than that of a random variable. The feature selection methods described here are designed based on this idea.

Assume that a set of *K* input-output pairs {(***x***_*k*_, *y*_*k*_)|*k* = 1, 2, ⋯ , *K*} is given, where ***x***_*k*_ = (*x*_1,*k*_, *x*_2,*k*_, ⋯ , *x*_*N,k*_), *x*_*i,k*_ is the value for the *i*-th input variable at the *k*-th observation, and *y*_*k*_ is the output value at the *k*-th observation. Then, the feature selection method (Kimura and Tokuhisa, 2020) tries to find all of the input variables relevant to the output according to the following procedure.

1. Construct a new training dataset {(***z***_*k*_, *y*_*k*_)|*k* = 1, 2, ⋯ , *K*} based on the given dataset {(***x***_*k*_, *y*_*k*_)|*k* = 1, 2, ⋯ , *K*}, where ***z***_*k*_ = (*x*_1,*k*_, *x*_2,*k*_, ⋯ , $x_{N,k},x_{1,k}^{pmt},x_{2,k}^{pmt},\cdots \;,$ $x_{N,k}^{pmt})$, and $x_{i,k}^{pmt}$ is the value for the *i*-th permuted input variable at the *k*-th observation. The values for the *i*-th permuted input variable in this algorithm, $x_{i,k}^{pmt}$'s, are obtained by randomly permuting those for the *i*-th original input variable, *x*_*i,k*_'s.
2. Train a random forest using the training dataset constructed in the step 1.
3. In order to statistically check whether or not input variables are relevant to the output, construct *N*_*RF*_ different random forests by repeating the steps 1 and 2.
4. When a value for $\frac{C_{i}-N_{RF}/2}{\sqrt{N_{RF}/4}}$ exceeds the α_*s*_-quantile of the standard normal distribution, conclude that the *i*-th input variable actually affects the output, where *C*_*i*_ is the number of random forests in which the importance score of the *i*-th original input variable is greater than that of the *i*-th permuted input variable. Note here that this study also uses a probability defined by $1-\frac{C_{i}}{N_{RF}}$ as a confidence value that the *i*-th input value actually affects the output.

To give the original and permuted input variables even chances of being selected for the splitting of the training examples, the feature selection method uses a slightly modified training algorithm for the random forest. See Kimura and Tokuhisa (2020) for more detailed information about the modification.

While the feature selection method described above is capable of detecting input variables that weakly affect the output, each irrelevant input variable is erroneously concluded to be relevant with a probability of about 0.5. In this study, we overcome the poor specificity of the feature selection method by constructing two other feature selection methods based on the same design concept (Kimura and Tokuhisa, 2020) and then combining all three of the methods together. To be specific, the two newly constructed feature selection methods respectively use Extra-Trees (Geurts et al., 2006) and VR-Trees (Liu et al., 2008), instead of the random forest, in the algorithm described above. Extra-Trees and VR-Trees are variants of the random forest. The method used to combine the three feature selection methods is described in the next section. While the original feature selection method uses a modified training algorithm for the random forest, note that the two newly constructed methods use the training algorithms for Extra-Trees and VR-Trees without any modification. In this paper, we refer to these three methods as the feature selection methods using the random forest, Extra-Trees, and VR-Trees, respectively.

### 3.2. Algorithm of the Combined Method

**Figure 1:**
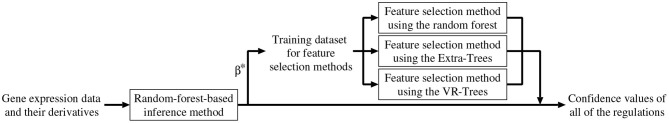
A framework of the proposed method.

As mentioned earlier, we combined the random-forest-based inference method (Kimura et al., 2019) with the feature selection methods described in the previous section. In addition to removing unpromising regulations, the combined method improves the confidence values of all of the candidate regulations. Below, we explain the algorithm of the combined method (see also Figure 1).

1. Set a counter *n* to 1.
2. Perform the random-forest-based inference method (Kimura et al., 2019) for the *n*-th subproblem, then obtain an approximation of the function *F*_*n*_ and a value for the parameter β_*n*_. Here, we represent them as ${\hat{F}}_{n}^{*}$ and $\beta_{n}^{*}$, respectively.
3. By applying ${\hat{F}}_{n}^{*}$ and $\beta_{n}^{*}$ to the Equation (3), compute the confidence value of the regulation of the *n*-th gene from the *m*-th gene, *C*_*n,m*_ (*m* = 1, 2, ⋯ , *N*, *m* ≠ *n*).
4. Construct a training dataset of input-output pairs,
  $$\begin{array}{l} \left\{\left({\text{X}}_{-n}{|}_{t_{k}},\frac{dX_{n}}{dt}{|}_{t_{k}}+\beta_{n}^{*}X_{n}{|}_{t_{k}}\right)|k=1,2,\cdots \;,K_{T}\right\}\\ \;\cup \left\{\left({\text{X}}_{-n}{|}_{s_{k}},\frac{dX_{n}}{dt}{|}_{s_{k}}+\beta_{n}^{*}X_{n}{|}_{s_{k}}\right)|k=1,2,\cdots \;,K_{S}\right\}, \end{array}$$
  and then apply the feature selection methods using the random forest, Extra-Trees, and VR-Trees to the constructed dataset. Note that the random-forest-based inference method used in this study trains models that consider the weight values, $w_{k}^{T}$'s and $w_{k}^{S}$'s, assigned to the given gene expression data. Therefore, our feature selection methods also consider these weight values when training the random forests, Extra-Trees, and VR-Trees used in these methods.
5. If one or more of the three feature selection methods conclude that the *m*-th gene does not regulate the *n*-th gene, set *C*_*n,m*_ to zero. In this study, a confidence value *C*_*n,m*_ equal to zero indicates that the proposed method infers no regulation of the *n*-th gene from the *m*-th gene. Otherwise, adjust the confidence value *C*_*n,m*_ according to
  $$C_{n,m}\leftarrow pC_{n,m}+\left(1-p\right)\min\left\{D_{n,m}^{RF},D_{n,m}^{ET},D_{n,m}^{VT}\right\},$$
  where *p* (0 ≤ *p* ≤ 1) is a mixing parameter. The mixing parameter represents the degree to which our combined method relies on the confidence values computed by the random-forest-based inference method. $D_{n,m}^{RF}$, $D_{n,m}^{ET}$, and $D_{n,m}^{VT}$ are the confidence values of the regulation of the *n*-th gene from the *m*-th gene, obtained from the feature selection methods using the random forest, Extra-Trees and VR-Trees, respectively. As mentioned in the section 3.1, the feature selection methods used in this study often falsely conclude an irrelevant input variable to be relevant. In this step, therefore, we adopt the worst estimate among the estimates obtained from the three feature selection methods in order to reduce the number of irrelevant regulations falsely concluded to be relevant.
6. *n* ← *n* + 1. If *n* ≤ *N*, return to the step 2.
7. Output all of the confidence values, i.e., *C*_*n,m*_'s (*m, n* = 1, 2, ⋯ , *N*, *m* ≠ *n*).

## 4. Experiments With Artificial Gene Expression Data

This section describes experiments conducted with artificial genetic network inference problems to evaluate the performance of the proposed method.

### 4.1. Analysis Using DREAM3 Data

To investigate the effect of the mixing parameter *p* on the performance of the proposed method, we first performed the experiment with a series of DREAM3 problems.

#### 4.1.1. Experimental Setup

The proposed method was applied to five artificial genetic network problems obtained from the DREAM3 *in silico* network challenges (http://dreamchallenges.org/): Ecoli1, Ecoli2, Yeast1, Yeast2, and Yeast3. The target networks of these problems consisted of 100 genes each (*N* = 100) and were designed based on actual biochemical networks.

Each problem used here contained both time-series and static expression data of all 100 genes. The time-series data were 46 datasets consisting of time-series of gene expression levels obtained by solving a set of differential equations on the target network, and were polluted by internal and external noise (Schaffter et al., 2011). The time-series datasets began from randomly generated initial values, and each gene in each set was assigned 21 observations, with time intervals of 10 between two adjacent observations. The static data consisted of wild-type, knockout and knockdown data. The wild-type data contained the steady-state gene expression levels of the unperturbed network. The knockout and knockdown data contained the steady-state expression levels of every single-gene knockout and every single-gene knockdown, respectively. When trying to solve the *n*-th subproblem corresponding to the *n*-th gene, however, we removed the static data of the knockout and the knockdown of the *n*-th gene. The number of measurements in the time-series experiment, *K*_*T*_, was therefore 46 × 21 = 966, while that of the steady-state experiment, *K*_*S*_, was 1 + 100 + 100 − 2 = 199. Noisy time-series data were provided as the observed data, so we smoothed them using a local linear regression (Cleveland, 1979), a data smoothing technique. The same local linear regression was used to estimate the time derivatives of the gene expression levels. The genetic network of 100 genes was inferred solely from the smoothed time-series of the gene expression levels, their estimated time derivatives, and the static gene expression data.

The number of trees in the random forest (*N*_*tree*_), the number of input variables to be considered in each internal node of each tree (*N*_*test*_), and the maximum height of each tree (*N*_*hmax*_) were set to 1, 000, $\left\lceil \frac{N-1}{3}\right\rceil$, and 32, respectively, according to the recommended parameter values for the random-forest-based inference method (Kimura et al., 2019). Because the parameter to be estimated, β_*n*_, was positive, we searched for an optimum value in a logarithmic space. The search area of log β_*n*_ was [−10, 5]. The inference method used in the proposed method must give values for the weight parameters for the gene expression data, i.e., $w_{k}^{T}$'s and $w_{k}^{S}$'s. The weight parameters for the measurements in each of the 46 time-series datasets were set at the values used by Kimura et al. (2019), namely, 0.6674 for the 10th measurement, 0.3348 for the 11th measurement, and 0.002174 for the last 10 measurements. The weight parameters for the other measurements in the time-series datasets and for the measurements in the static dataset were set to 1.0 and 1.1, respectively.

The number of random forests constructed (*N*_*RF*_), the number of trees in each random forest, and the significance level of the statistical test (α_*s*_) were set to 100, 100, and 0.01, respectively, for the feature selection method using the random forest, as well as for the feature selection methods using Extra-Trees and VR-Trees. Again, the recommended values were used for the other parameters for the feature selection methods: the numbers of input variables to be considered in each internal node of each tree in the random forest and in Extra-Trees were set to $\left\lceil \frac{N-1}{3}\right\rceil \times 2$ and (*N* − 1) × 2, respectively, and α, the parameter that controls the probability that the deterministic test-selection will be selected over the random test-selection in VR-Trees, was set to 0.5.

Another parameter, namely, the mixing parameter *p*, must also be assigned a value in the proposed method. In this study, we investigated how the parameter *p* affected the performance of our method by running a series of experiments with different mixing parameter values. As the proposed method is a stochastic algorithm, we applied the method with each of the parameter settings to each of the five problems ten times.

#### 4.1.2. Results

We tested the performance of the proposed method using the area under the recall-precision curve (AURPC), a performance measure that increases from 0 to 1 as the numbers of false-positive and false-negative regulations decrease. The recall-precision curve of an algorithm was obtained by checking the recalls and precisions. The recall and the precision are defined as

$$\text{recall}=\frac{TP}{TP+FN},\text{ precision}=\frac{TP}{TP+FP},$$

where *TP*, *FP*, and *FN* are the numbers of true-positive, false-positive, and false-negative regulations, respectively. The recall and precision were computed by constructing a network of regulations whose confidence values exceeded a threshold, and then comparing it with the target network. Note that the proposed method assigns confidence values to all of the candidate regulations. Next, the recall-precision curve of the algorithm was obtained by changing the threshold for the confidence value. Auto-regulations/auto-degradations were disregarded in the evaluation of the performance.

Table 1 lists the AURPCs of the proposed method with different mixing parameter values in the five problems. The table also shows the performance of the random-forest-based inference method (Kimura et al., 2019), a method equivalent to that proposed here without the feature selection. As described in the section 3.2, the proposed method removes unpromising candidate regulations and then adjusts the confidence values of the remaining the candidates. When the mixing parameter *p* is set to 1.0, however, our method omits this adjustment of the confidence values. The experimental results thus show that the removal of the unpromising candidate regulations improves the performance of the inference method only to a slight degree. Note that our method removed 268.4, 235.8, 208.9, 73.0, and 107.6 candidate regulations, on average, in Ecoli1, Ecoli2, Yeast1, Yeast2, and Yeast3, respectively. Given that Ecoli1, Ecoli2, Yeast1, Yeast2, and Yeast3 have *N* × (*N* − 1) = 9, 900 candidate regulations each, and 125, 119, 166, 389, and 551 actual regulations, respectively, we see that the numbers of regulations removed by the proposed method were very small. Hence, the limited improvement in the performance might be explained by the small number of unpromising candidate regulations removed in the five problems solved.

**Table 1:** The performance of the proposed method with different values for the mixing parameter *p* on the DREAM3 problems.

		**Ecoli1**	**Ecoli2**	**Yeast1**	**Yeast2**	**Yeast3**
		**AVG**	**AVG**	**AVG**	**AVG**	**AVG**
		**± STD**	**± STD**	**± STD**	**± STD**	**± STD**
Proposed method	(*p* = 1.0)	0.41910	0.54478	0.50084	0.39486	0.31297
		±0.00390	±0.00586	±0.00287	±0.00344	±0.00224
Proposed method	(*p* = 0.9)	0.42143	0.54539	0.50594	0.40047	0.32093
		±0.00378	±0.00563	±0.00289	±0.00370	±0.00221
Proposed method	(*p* = 0.8)	0.42307	0.54607	0.50825	0.40261	0.32234
		±0.00380	±0.00530	±0.00301	±0.00371	±0.00245
Proposed method	(*p* = 0.7)	0.42422	0.54674	0.50984	0.40384	0.32256
		±0.00395	±0.00523	±0.00314	±0.00377	±0.00263
Proposed method	(*p* = 0.6)	0.42493	0.54736	0.51055	0.40446	0.32223
		±0.00420	±0.00537	±0.00347	±0.00384	±0.00272
Proposed method	(*p* = 0.5)	0.42532	0.54772	0.51060	0.40419	0.32151
		±0.00437	±0.00542	±0.00395	±0.00393	±0.00278
Proposed method	(*p* = 0.4)	0.42520	0.54767	0.50996	0.40321	0.32054
		±0.00465	±0.00579	±0.00446	±0.00393	±0.00291
Proposed method	(*p* = 0.3)	0.42410	0.54689	0.50856	0.40179	0.31975
		±0.00533	±0.00658	±0.00472	±0.00378	±0.00297
Proposed method	(*p* = 0.2)	0.42216	0.54514	0.50655	0.40059	0.31922
		±0.00663	±0.00766	±0.00489	±0.00386	±0.00293
Proposed method	(*p* = 0.1)	0.42034	0.54344	0.50332	0.40046	0.31881
		±0.00757	±0.00813	±0.00507	±0.00390	±0.00265
Proposed method	(*p* = 0.0)	0.07094	0.07486	0.09892	0.13139	0.13949
		±0.00195	±0.00206	±0.00252	±0.00232	±0.00284
Random-forest-based inference method		0.41918	0.54477	0.50083	0.39482	0.31291
(Kimura et al., 2019)		±0.00388	±0.00586	±0.00285	±0.00344	±0.00223

*The performance of the random-forest-based inference method (Kimura et al., 2019) is also shown. AVG and STD represent the averaged AURPC and its standard deviation, respectively*.

When the mixing parameter *p* is set to 0.0, on the other hand, the proposed method outputs the confidence values of the regulations computed only on the basis of the values provided by the feature selection methods. The experimental results of our method with *p* = 0.0 indicate that the confidence values computed by the feature selection methods are unreliable. As the table shows, however, we can improve the performance of the proposed method by combining the confidence values computed by the random-forest-based inference method with those computed by the feature selection methods. Our method seems to perform at its best when the parameter *p* is set to around 0.5. The standard deviations of the AURPCs, on the other hand, widened as the value for parameter *p* fell from 0.9 to 0.1. As a result, the network inferred by the proposed method with a smaller parameter *p* was likely to be of a lower quality than that inferred by the method without the feature selection methods. In the remaining experiments in this study, we thus set the mixing parameter *p* to 0.9. The networks inferred by the proposed method with *p* = 0.9 were better than those inferred by the method without the feature selection in 49 of the 50 (= 5 × 10) trials performed on the DREAM3 problems.

The proposed method has a much higher computational cost than the random-forest-based inference method (Kimura et al., 2019), as the random forest, Extra-Trees, and VR-Trees must be trained many times. As described earlier, we divided the inference problem of a genetic network consisting of 100 genes into 100 subproblems. While the random-forest-based inference method (Kimura et al., 2019) required an average of 30.3 min to solve a single subproblem, the proposed method required an average of 127.9 min to solve a subproblem on the same workstation (Xeon Gold 6150 2.7GHz). Though inconvenient, we do not see high computational cost of the proposed method as a hindrance to our study, given that our primary aim is to extract as much useful information as possible from a limited amount of gene expression data. Moreover, the computation time required by our method can be easily shortened by performing the calculations in parallel.

### 4.2. Analysis Using DREAM4 Data

Our next step was to compare the proposed method with the other genetic network inference methods on the DREAM4 problems.

#### 4.2.1. Experimental Setup

For our next experiment, we applied the proposed method to five problems from the DREAM4 *in silico* network challenges. Similar to the DREAM3 problems, the target networks in these problems consisted of 100 genes, and were designed based on actual biochemical networks. These networks were described using a model identical to that of the DREAM3 networks (Schaffter et al., 2011).

Each problem contained both the time-series and static expression data of all 100 genes. The time-series data were 10 datasets of time-series of gene expression levels. Each dataset consisted of the expression levels at 21 time points, and was polluted by internal and external noise. A dataset was constructed by applying a perturbation to the network at the first time point and removing the perturbation at the 11-th time point. The perturbation affected the transcription rates of a different set of several genes in each dataset. The static data consisted of wild-type, knockout, and knockdown data.

To take the perturbations into account explicitly, we added 10 elements to the gene expression data, each corresponding to one of the perturbations. The *i*-th added element had a value of 1 for the measurements between the 1st and 10th time points in the *i*-th time-series dataset generated by adding the *i*-th perturbation, and a value of 0 for the other measurements. The number of elements, *N*, was therefore 100 + 10 = 110. When trying to solve the *n*-th subproblem corresponding to the *n*-th gene, we also removed the static data of the knockout and the knockdown of the *n*-th gene. The numbers of measurements of the time-series and steady-state experiments, i.e., *K*_*T*_ and *K*_*S*_, were thus 10 × 21 = 210 and 1 + 100 + 100 − 2 = 199, respectively. The local linear regression (Cleveland, 1979) was used to smooth the given time-series data and to estimate the time derivatives of the gene expression levels. We inferred a genetic network using only the smoothed time-series of the gene expression levels, their estimated time derivatives, and the static gene expression data.

The 6th, 7th, 8th, 9th, and 10th measurements in each of the time-series datasets were all assigned weight values of 0.2 (Kimura et al., 2019). The 17th, 18th, 19th, 20th, and 21th measurements were all assigned weight values of 0.02. The 4th, 5th, 15th, and 16th measurements were assigned weight values of 0.7333, 0.4667, 0.6733, and 0.3466, respectively. The values for the remaining $w_{k}^{T}$'s and for $w_{k}^{S}$'s were set to 1.0 and 1.1, respectively. As described in the section 4.1.2, the mixing parameter *p* was set to 0.9. The other experimental conditions were unchanged from those used in the section 4.1.

#### 4.2.2. Results

We also used the area under the recall-precision curve (AURPC) to quantify the performance of the inference method in this experiment. Although we inferred the regulations of the 100 genes from these genes and the 10 additional elements representing 10 perturbations, we disregarded the regulations of the genes from the additional elements for the evaluation of the performance. Auto-regulations/auto-degradations were also disregarded in the evaluation of the performance. Table 2 shows the AURPCs of the proposed method on the five problems, along with the AURPCs of the original random-forest-based inference method (Kimura et al., 2019), dynGENIE3 (Huynh-Thu and Geurts, 2018), MCZ (Greenfield et al., 2010), a combination of dynGENIE3 and MCZ, and iRafNet (Petralia et al., 2015). The AURPCs of dynGENIE3, MCZ, and the combination of dynGENIE3 and MCZ are taken from Huynh-Thu and Geurts (2018), while the AURPCs of iRafNet are taken from Petralia et al. (2015).

**Table 2:** The AURPCs of the proposed method with *p* = 0.9 on the DREAM4 problems.

		**Network1**	**Network2**	**Network3**	**Network4**	**Network5**
		**AVG**	**AVG**	**AVG**	**AVG**	**AVG**
		**± STD**	**± STD**	**± STD**	**± STD**	**± STD**
Proposed method	(*p* = 0.9)	0.44629	0.31188	0.35118	0.35700	0.28935
		±0.00351	±0.00364	±0.00369	±0.00366	±0.00399
Random-forest-based inference method		0.42797	0.28656	0.33930	0.34079	0.27199
(Kimura et al., 2019)		±0.00312	±0.00300	±0.00397	±0.00347	±0.00415
dynGENIE3		0.34	0.22	0.32	0.34	0.22
(Huynh-Thu and Geurts, 2018)		—	—	—	—	—
MCZ		0.48	0.38	0.38	0.36	0.17
(Greenfield et al., 2010)		—	—	—	—	—
dynGENIE3 + MCZ		0.60	0.43	0.47	0.52	0.37
		—	—	—	—	—
iRafNet		0.552	0.337	0.414	0.421	0.298
(Petralia et al., 2015)		—	—	—	—	—

*The table also shows the performances of the random-forest-based inference method (Kimura et al., 2019), dynGENIE3 (Huynh-Thu and Geurts, 2018), MCZ (Greenfield et al., 2010), a combination of dynGENIE3 and MCZ, and iRafNet (Petralia et al., 2015)*.

As the table illustrates, the use of the feature selection methods improved the quality of the inferred network. The improvements brought about by the feature selection methods were larger than the improvements obtained in the experiment performed in the section 4.1. The better performance obtained might have partly stemmed from the larger number of unpromising regulations removed by the proposed method on the DREAM4 problems. Our method removed an average of 2075.6, 1676.1, 1797.8, 1652.8, and 1559.9 regulations from 100 × 109 = 10900 candidate regulations in Network1, Network2, Network3, Network4, and Network5, respectively.

The proposed method, however, failed to outperform the other inference methods in some cases, as the table shows. Note however that dynGENIE3 and iRafNet are both designed based on the random forest. As such, we could modify these inference methods to improve the performance by applying the proposed idea. Remember also that, when using MCZ, we must provide static data for every single-gene knockout if we are to obtain a reasonable genetic network. The use of static data for every single-gene knockout might partly explain the excellent performance of the combination of dynGENIE3 and MCZ. The excellent performance of iRafNet seems to stem from a similar cause. Data of this type, however, are difficult to measure, which puts a limit to their practical use.

## 5. Analysis of Biological Gene Expression Data

In the final experiment of this study, we used the proposed method to analyze actual gene expression data.

### 5.1. Experimental Setup

In this experiment, we analyzed the expression data of 11 immediate early genes related to transcription, i.e., ATF3, EGR1, EGR2, EGR3, ETS2, FOS, FOSB, FOSL1, JUN, JUNB, and MYC. The time-series and static gene expression levels were obtained from FANTOM5 data (http://fantom.gsc.riken.jp/5/) (FANTOM Consortium et al., 2014). The time-series datasets consisted of sets of expression levels of the genes measured in Saos-2, MCF-7, ARPE-19, lymphatic endothelial, mesenchymal stem, and aortic smooth muscle cells at successive time points after several kinds of external stimuli were applied. Table 3 presents detailed information on the time-series datasets used in this study. Two types of static data were used for the experiment. The first were sets of gene expression levels for the Saos-2 and mesenchymal stem cells introduced as untreated controls. The second were the measurements taken at time 0 in the respective time-series datasets. The numbers of measurements contained in the time-series and static data in this experiment, *K*_*T*_ and *K*_*S*_, were 11 + 16 + 16 + 13 + 16 + 10 + 10 + 10 = 102 and 2 + 8 = 10, respectively. Eight elements corresponding to the stimuli applied to the cells were added to the gene expression data, in order to take the external stimuli explicitly into account: “ascorbic acid and BGP,” “EGF1,” “HRG,” “TGF-β and TNF-α,” “VEGF,” “IBMX, DEX and insulin,” “FGF-2,” and “IL-1B.” An added element had a value of 1 for the measurements in the time-series dataset obtained by applying the stimulus corresponding to the element, and a value of 0 for the other measurements. The total number of elements, *N*, was therefore 11 + 8 = 19. By applying the proposed method to the gene expression data described here, we inferred regulations of the 11 selected genes from both the 11 genes and the 8 additional elements. These gene expression data were also analyzed in Kimura et al. (2019).

**Table 3:** The measurement conditions of the time-series datasets used in this study.

**Cell name**	**Stimulus**	**Measured time (min.)**
Saos-2 cells	Ascorbic acid	0, 15, 30, 45, 60, 80, 100, 120, 150,
	and BGP	180, 240
MCF-7 cells	EGF1	0, 15, 30, 45, 60, 80, 100, 120, 150,
		180, 210, 240, 300, 360, 420, 480
MCF-7 cells	HRG	0, 15, 30, 45, 60, 80, 100, 120, 150,
		180, 210, 240, 300, 360, 420, 480
ARPE-19 cells	TGF-β and	0, 15, 30, 45, 60, 80, 100, 120, 150,
	TNF-α	180, 210, 240, 300
Lymphatic	VEGF	0, 15, 30, 45, 60, 80, 100, 120, 150,
endothelial cells		180, 210, 240, 300, 360, 420, 480
Mesenchymal	IBMX, DEX	0, 15, 30, 45, 60, 80, 100, 120, 150,
stem cells	and insulin	180
Aortic smooth	FGF-2	0, 15, 30, 45, 60, 120, 180, 240, 300,
muscle cells		360
Aortic smooth	IL-1B	0, 15, 30, 45, 60, 120, 180, 240, 300,
muscle cells		360

The following weight values for the expression data were determined according to Kimura et al. (2019). The weight values corresponding to the 11th, 12th, 13th, 14th 15th, and 16th measurements in the time-series dataset of the lymphatic endothelial cells were set to 0.75, 0.5, 0.25, 0.25, 0.25, and 0.25, respectively. The weight values for the 8th, 9th, 10th, and 11th measurements in the time-series dataset of the Saos-2 cells, and for the 7th, 8th, 9th, and 10th measurements in the two time-series datasets of the aortic smooth muscle cells, were set to 0.8333, 0.6667, 0.5, and 0.5, respectively. The weight values for the two measurements in the steady-state experiments with the Saos-2, MCF-7, mesenchymal stem, and aortic smooth muscle cells were set to 0.55. The weight values for the other measurements in the time-series and static datasets were set to 1.0 and 1.1, respectively. The other experimental settings were identical to those used in the previous experiment.

### 5.2. Results

Table 4 lists the top 20 regulations with respect to the confidence values computed by the proposed method. The correct structure of the target network, however, is still unknown. We thus compared the inferred regulations with those obtained from the STRING database (https://string-db.org/) (Szklarczyk et al., 2014) of protein-protein interactions. The comparison results suggest that 13 of the 20 regulations (boldface font in the table) are reasonable, as the interactions between the proteins corresponding to the genes have been confirmed in human and/or other species. Moreover, the regulation of ATF3 from the external stimulus “TGF-β and TNF-α” (italic font in the table) seems to be reasonable because TGF-β has been confirmed to induce ATF3 (Yin et al., 2010).

**Table 4:** The top 20 regulations ranked by the confidence values computed by the proposed method.

**Rank**	**Result from original data**	**Result from modified data**
1	**EGR1** ← **FOS**	**EGR1** ← **FOS**
2	**EGR2** ← **FOS**	*FOS ← HRG*
3	*ATF3 ← TGF-β and TNF-α*	*ATF3 ← TGF-β and TNF-α*
4	**JUNB** ← **FOSB**	*EGR2 ← HRG*
5	EGR3 ← EGR2	**JUNB** ← **FOSB**
6	**FOSL1** ← **ATF3**	EGR3 ← EGR2
7	**MYC** ← **FOS**	**EGR3** ← **FOS**
8	EGR1 ← EGR2	**FOSL1** ← **ATF3**
9	**EGR3** ← **FOS**	**EGR2** ← **FOS**
10	**FOSB** ← **JUNB**	EGR1 ← EGR2
11	**JUNB** ← **EGR2**	**MYC** ← **FOS**
12	EGR3 ← EGR1	**JUNB** ← **EGR2**
13	**FOS** ← **EGR2**	EGR3 ← EGR1
14	ETS2 ← EGR2	**FOSB** ← **JUNB**
15	**JUN** ← **FOSB**	**JUN** ← **VEGF**
16	**EGR2** ← **MYC**	ETS2 ← EGR2
17	**JUN** ← **VEGF**	**JUN** ← **FOSB**
18	EGR2 ← EGR1	FOSL1 ← FOSB
19	FOSL1 ← FOSB	**FOSB** ← **EGR2**
20	**FOSB** ← **EGR2**	**ATF3** ← **JUN**

*The rankings are obtained from an analysis of original data identical to those of Kimura et al. (2019), and the modified data constructed by considering the decomposition of the chemical compounds used for the stimulation of the cells. The regulations written in boldface and italic fonts have reportedly been confirmed in human and/or other species and are accordingly assumed to be reasonable*.

The proposed method, on the other hand, concluded that 28 candidate regulations were unpromising, and set their confidence values to zero. While the regulations of EGR1 from the external stimuli “FGF-2” and “IL-1B” were among the 28 removed regulations, the protein-protein network obtained from the STRING database suggested that these two regulations should not be removed. As described in the section 5.1, this study represented the existence and absence of an external stimulus as 1 and 0, respectively. This simple representation might help to explain the erroneous conclusion that the two regulations just mentioned were unpromising.

Our next step, therefore, was to obtain a more reasonable genetic network by making the representation of the external stimuli more realistic. To do so, we first had to consider the decomposition of the chemical compounds used for stimulating the cells. When preparing the gene expression data, we set the value for each of the 8 added elements corresponding to the external stimuli to $0.9^{\frac{t}{48}}$, instead of 1, where *t* was the time (min.) elapsed after the stimulation of the cells. We then applied the proposed method to the modified gene expression data. Table 4 also shows the top 20 regulations ranked by the confidence values obtained in the additional experiment with the modified data. To check the effect of the modification of the data, we compared the inferred regulations with those contained in the protein-protein network obtained from the STRING database. The comparison indicated that 12 of the 20 regulations were reasonable (boldface font in the table), as the interactions between the corresponding proteins were reportedly confirmed. We could also conclude, for the reason mentioned previously, that the regulation of ATF3 from the external stimulus “TGF-β and TNF-α” was reasonable. The regulations of FOS and EGR2 from the external stimulus “HRG” (italic font in the table) appeared to be reasonable as well, given the suggestion from Yuan et al. (2008) and Martine-Moreno et al. (2017) that these regulations existed. In the top 20 regulations inferred in the additional experiment, the number of reasonable regulations was larger, and the ranks of the unreasonable regulations seemed to be slightly lower. The regulations of EGR1 from the external stimuli “FGF-2” and “IL-1B,” meanwhile, were erroneously removed in the experiment with the original gene expression data, as mentioned earlier. These two regulations remained in the inferred regulations in this additional experiment, although the number of removed regulations decreased to 18.

As mentioned earlier, the improvement in performance brought about by combining the random-forest-based inference method with the feature selection methods is often small. In the experiments in this section, therefore, the top 20 regulations obtained by the proposed method were completely identical to those of the original random-forest-based method (Kimura et al., 2019). Moreover, the numbers of regulations removed by the proposed method were also modest. By comparing the removed regulations with those now known, however, we can check the validity of the inferred network. This feature of the proposed method could be useful, when we try to analyze actual gene expression data.

## 6. Conclusion

Several random-forest-based inference methods have been proposed. While these methods show promise, they are only of use in ranking all of the candidate regulations by assigning them confidence values. They are of no use in removing unnecessary regulations. In this study, we propose a new method to remove unpromising candidate regulations by combining the random-forest-based inference method (Kimura et al., 2019) with the original feature selection method (Kimura and Tokuhisa, 2020) and two modifications of that method. By using the outputs from the feature selection methods, the proposed method also adjusts the confidence values of the candidate regulations. Numerical experiments performed with artificial gene expression data showed that the combination of the inference method with the feature selection methods slightly improved the quality of the inferred genetic network. Though its computational cost is high, we believe that the proposed method is useful for our chief purpose of extracting as much useful information as possible from a limited amount of gene expression data. Through experiments with actual data, we showed that the removal of unpromising regulations is a useful feature for confirming the validity of an inferred genetic network. The number of regulations removed by the proposed method, however, was often very small. In future work, we plan to search for strategies to detect larger numbers of unpromising regulations.

## Data Availability Statement

The raw data supporting the conclusions of this article will be made available by the authors, without undue reservation.

## Author Contributions

SK developed the method and performed the experiments. RF and MT designed some parts of the proposed algorithm. MO supervised the biological aspect of this work. All authors read and approved the manuscript.

## Conflict of Interest

The authors declare tha the research was conducted in the absence of any commercial or financial relationships that could be construed as a potential conflict of interest.
