# Supplementary material for: Inference of Genetic Networks From Time-Series and Static Gene Expression Data: Combining a Random-Forest-Based Inference Method With Feature Selection Methods
Source: Front Genet. 2020 Dec 15;11:595912. doi: 10.3389/fgene.2020.595912 (PMC7770182; doi:10.3389/fgene.2020.595912)
Supplement: Supplementary file 2 [file Data_Sheet_1.PDF]

Supporting Information:
Inference of genetic networks using random forests: using feature selection methods to detect unpromising regulations

Shuhei Kimura *et al.*
kimura@tottori-u.ac.jp

1 Inference Methods with Existing Feature Selection Methods

In this section, we compared the proposed method with the methods that combine the random-forest-based inference method with the existing feature selection methods.

1.1 Existing feature selection methods

This study combined the inference method [2] with each of the following feature selection methods, i.e., the forward selection and the backward elimination [1]. They are simple but widely used methods.

1.1.1 Forward selection

Assume that, amnong N input variables, denoted here as $X_1, X_2, \cdots, X_N$, we try to find input variables that actually affect the output. Then, the forward selection method try to find input variables according to the following procedure.

1. Initialize a selected feature set F_s and a candidate feature set F_c to ϕ and $\{X_1, X_2, \cdots, X_N\}$, respectively. $F_s^* \leftarrow F_s$.
2. For each $X \in F_c$, evaluate the performance of the random forest with input variables $F_s \cup X$. In this study, we use the leave-one-out cross-validation [3] to evaluate the performance of the random forest. Then, select the best-performing variable, denoted here by X^*.
3. $F_s \leftarrow F_s \cup X^*$ and $F_c \leftarrow F_c \setminus X^*$. If the random forest with the input variables F_s performs better than that with the input variables F_s^*, then $F_s^* \leftarrow F_s$. If $F_c = \phi$, output F_s^* and then stop. Otherwise, return to the step 2.

1.1.2 Backward elimination

Following are the steps of the backward elimination method used in this study.

1. Initialize a selected feature set F_s to $\{X_1, X_2, \cdots, X_N\}$. Evaluate the performance of the random forest with the input variables F_s. In this algorithm, we also use the leave-one-out cross-validation to evaluate the performance of the random forest. $F_s^* \leftarrow F_s$.

2. For each $X \in F_s$, evaluate the performance of the random forest with the input variables $F_s \setminus X$. Then, select the best-performing variable, X^*.

3. $F_s \leftarrow F_s \setminus X^*$. If the random forest with the input variables F_s performs better than that with F_s^*, then $F_s^* \leftarrow F_s$. If $F_s = \phi$, output F_s^* and then stop. Otherwise, return to the step 2.

1.2 Combining random-forest-based inference method with feature selection method

This study simply combined the inference method [2] with each of the feature selection methods described above according to the following procedure.

1. Set a counter n to 1.

2. Perform the random-forest-based inference method [2] for the n-th subproblem, and then obtain the approximation of the function F_n and the value for the parameter β_n. This study represents them as $\hat{F}_n^*$ and β_n^*, respectively. Then, compute the confidence value of the regulation of the n-th gene from the m-th gene, $C_{n,m}$ ($m = 1, 2, \cdots, N$, $m \neq n$).

3. Construct a training dataset of input-output pairs,

$$\left\{ \left(\mathbf{X}_{-n}|_{t_k}, \left.\frac{dX_n}{dt}\right|_{t_k} + \beta_n^* \, X_n|_{t_k} \right) \middle| k = 1, 2, \cdots, K_T \right\}$$
$$\cup \left\{ \left(\mathbf{X}_{-n}|_{s_k}, \left.\frac{dX_n}{dt}\right|_{s_k} + \beta_n^* \, X_n|_{s_k} \right) \middle| k = 1, 2, \cdots, K_S \right\},$$

   and then apply either of the feature selection methods described above.

4. If the feature selection method applied concludes that the m-th gene does not regulate the n-th gene, set $C_{n,m}$ to zero.

5. $n \leftarrow n + 1$. If $n \leq N$, return to the step 2.

6. Output all of the confidence values, i.e., $C_{n,m}$'s ($m, n = 1, 2, \cdots, N$, $m \neq n$).

1.3 Experiment on Random Networks

In this experiment, we compared the proposed method with the inference methods described in the previous section.

1.3.1 Experimental setup

We used the Vohradský's model[4] consisting of 30 genes ($N = 30$) as target networks. The Vohradský's model is a set of differential equations of the form

$$\frac{dX_n}{dt} = \frac{k_{1n}}{1 + \exp\left(-b_n - \sum_{m=1}^{N} w_{n,m} X_m\right)} - k_{2n} X_n, \qquad (n = 1, 2, \cdots, N). \tag{1}$$

According to the reference [2], we generated the target networks of different structures by changing the model parameters. When trying to determine the model parameters corresponding to the n-th gene, we randomly chose

Table 1: The performances of the proposed method, the random-forest-based inference method [2], the methods that combine the inference method with the existing feature selection methods. AVG and STD represent the averaged AURPC and its standard deviation, respectively. '# removed regulations' represents the number of the regulations that were concluded as unpromising ones.

	AVG $\pm$ STD	# removed regulations
Proposed method ($p = 0.9$)	0.68411 ± 0.04695	20.3
Random-forest-based inference method [2]	0.68207 ± 0.04622	—
Combination of inference method [2] with forward selection	0.64561 ± 0.06414	616.8
Combination of inference method [2] with backward elemination	0.62723 ± 0.05629	620.1

an integer k from a power-law distribution with a cutoff of 5. Then, k genes were randomly selected from all of the genes contained in the network. The parameters $w_{n,m}$'s corresponding to the regulations of the n-th gene from the selected genes were randomly chosen from $[-10.0, -5.0] \cup [5.0, 10.0]$, and the remaining $w_{n,m}$'s were set to 0.0. We also randomly selected the parameters k_{1n} and k_{2n} from $[1.0, 3.0]$. The parameter b_n was set to $-\sum_{m=1}^{N} w_{n,m}$. We constructed 10 genetic network inference problems with different target networks.

Ten time-series datasets were generated by solving a set of the differential equations (1) on each of the target models. The initial values of these sets were selected randomly from $[0.0, 3.0]$. Each dataset consisted of the expression levels at 21 time points with time intervals of 0.2. As the static data, we constructed steady-state gene expression levels of wild-type and every single-gene knockout. The measurement noise was simulated by adding 10% Gaussian noise to the computed gene expression data. We did not use the steady-state gene expression levels of the knockout of the n-th gene when trying to analyze the n-th gene. The numbers of measurements contained in the time-series and static data, i.e., K_T and K_S, were therefore $10 \times 21 = 210$ and $1 + 100 - 1 = 100$, respectively.

We also set the weight parameters according to the reference [2]: We set the weight values for the last 6 measurements in each of the time-series datasets to 0.01667; The weight values for the 14th and 15th measurements in each of the time-series datasets were set to 0.6722 and 0.3444, respectively, and the remaining weight parameters for the time-series datasets and for the static dataset were set to 1.0 and 1.1, respectively. For the parameters of the proposed method, this study used their recommended values.

1.3.2 Results

Table 1 shows the AURPCs of the proposed method, the random-forest-based inference method [2], the method that combines the inference method [2] with the forward selection, and the method that combines the inference method [2] with the backward elimination. As the table shows, by combining the inference method with the existing feature selection method, we can remove lots of regulations. However, as some of them should not be removed, the performances of the methods that combine the inference method with the existing feature selection methods were worse. The degradation of the performance would be caused by a reason that the purpose of the existing feature selection methods is not detect all of the input variables that actually affect the output but to find input variables that make the predicting performance of the model the best. Although the numbers of regulations removed by the proposed method were much smaller, on the other hand, it did not remove any unnecessary regulations. Thus, the quality of the networks inferred by the proposed method was always better.

2 Supporting information for the section 4

Samples of the recall-precision curves obtained by the proposed method with $p = 0.9$ and the random-forest-based inference method [2] on the DREAM3 and DREAM4 problems are shown in Figures 1 and 2, respectively.

References

[1] I. Guyon and A. Elisseeff, An introduction to variable and feature selection, J. of Machine Learning Research 3 (2003) 1157–1182.

[2] S. Kimura, M. Tokuhisa and M. Okada, Inference of genetic networks using random forests: Assigning different weights for gene expression data, J. of Bioinformatics and Computational Biology 17 (2019) 1950015.

[3] R. Kohavi, A study of cross-validation and bootstrap for accuracy estimation and model selection, Proc. of 14th Int. Joint Conf. on Artificial Intelligence (1995) 1137–1143.

[4] J. Vohradský, Neural network model of gene expression, FASEB J. 15 (2001) 846–854.

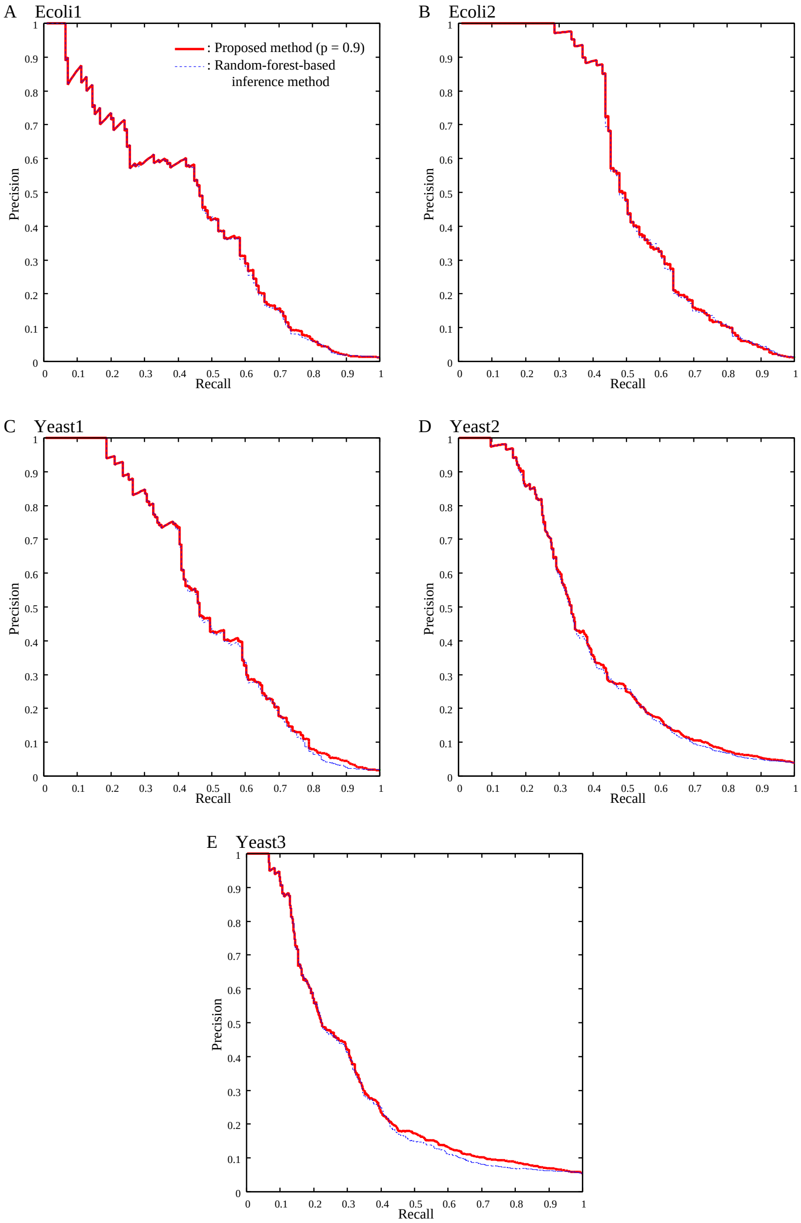

Figure 1: Samples of the recall-precision curves of the proposed combined method ($p = 0.9$) and the random-forest-based inference method on the DREAM3 problems of (A) Ecoli1, (B) Ecoli2, (C) Yeast1, (D) Yeast2, and (E) Yeast3. Solid and dotted lines represent the performance of the proposed combined method and the random-forest-based inference method, respectively.

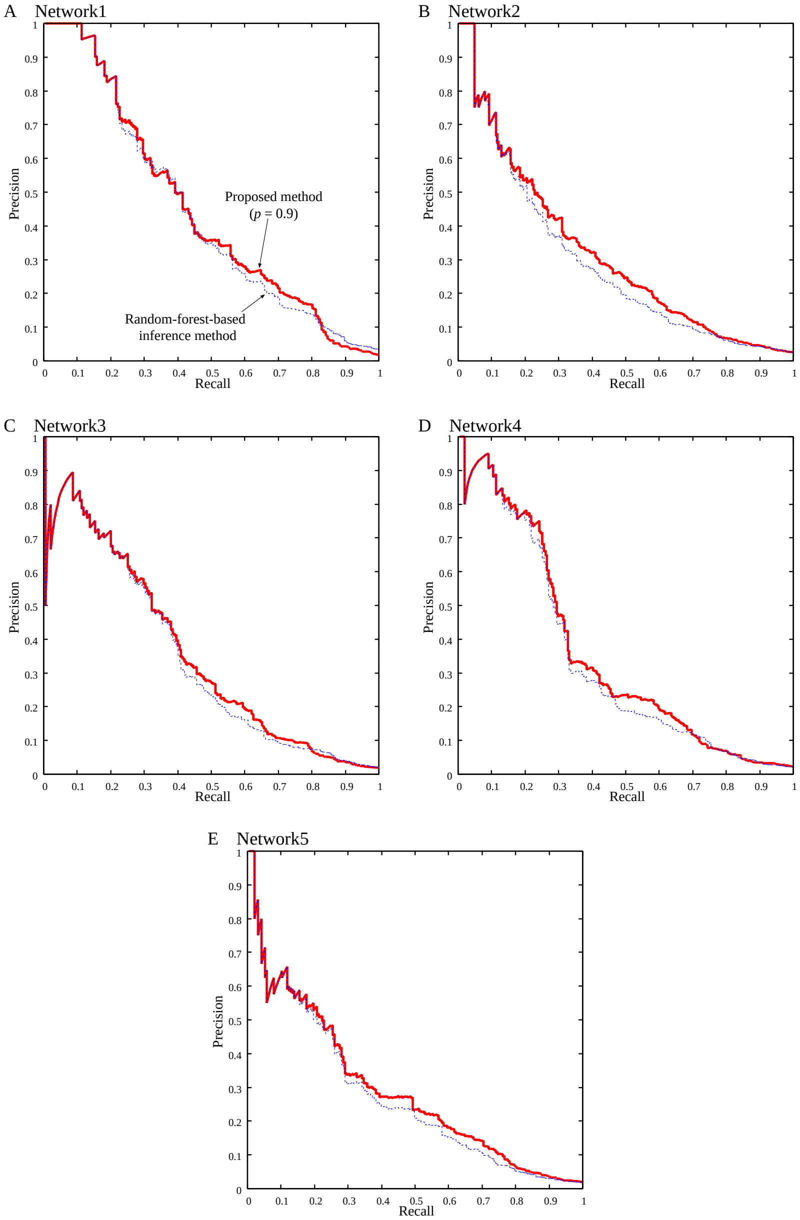

Figure 2: Samples of the recall-precision curves of the proposed combined method ($p = 0.9$) and the random-forest-based inference method on the DREAM4 problems of (A) Network1, (B) Network2, (C) Network3, (D) Network4, and (E) Network5. Solid and dotted lines represent the performance of the proposed combined method and the random-forest-based inference method, respectively.
